# New cases of Glucose-6-Phosphate Dehydrogenase deficiency in Pulmonary Arterial Hypertension

**DOI:** 10.1371/journal.pone.0203493

**Published:** 2018-08-30

**Authors:** Sergey Kurdyukov, Cody A. Eccles, Ankit A. Desai, Manuel Gonzalez-Garay, Jason X.-J. Yuan, Joe G. N. Garcia, Olga Rafikova, Ruslan Rafikov

**Affiliations:** 1 Division of Endocrinology, Department of Medicine, College of Medicine, The University of Arizona, Tucson, Arizona, United States of America; 2 Division of Cardiology, Department of Medicine, College of Medicine, The University of Arizona, Tucson, Arizona, United States of America; 3 Division of Translational and Regenerative Medicine, Department of Medicine, College of Medicine, The University of Arizona, Tucson, Arizona, United States of America; 4 Division of Pulmonary and Critical Care Medicine; Department of Medicine, College of Medicine, The University of Arizona, Tucson, Arizona, United States of America; Stanford University, UNITED STATES

## Abstract

Pulmonary Arterial Hypertension (PAH) is a fatal disorder with limited treatment options and reduced life expectancy after diagnosis. Complex genetic backgrounds in PAH complicates identification of causative mutations that is essential for an understanding of the disease diagnostics and etiology especially for idiopathic PAH (iPAH). Hemolysis has been implicated as contributing to the pathobiology of PAH. Glucose-6-Phosphate Dehydrogenase (G6PD) expression and activity define erythrocyte’s antioxidant capacity, and its decrease contributes to erythrocyte fragility. As G6PD deficiency was previously reported in a limited number of PAH cases, we tested whether iPAH patients exhibit underlying G6PD alterations in erythrocytes. A cohort of 22 PAH patients and 8 non-PAH patients were recruited for this study. DNA isolated from Peripheral Blood Mononuclear Cells (PBMC) was used for detection of mutations in the coding region of the *G6PD* gene. RNA isolated from PBMC was used for determination of *G6PD* mRNA expression level. G6PD activity was measured in Red Blood Cell (RBC) pellets. Three patients had missense mutations in G6PD (Val291Met, Asn126Asp, Asp194Glu), however, only one mutation (Val291Met) results in a severe G6PD deficiency. A single patient with mutation (Asn126Asp) showed a 21% decrease in G6PD activity, two subjects showed G6PD deficiency without mutations, and one patient had a decreased level of G6PD mRNA and reduced enzyme levels. This study demonstrates that a moderate decrease in G6PD activity is associated with PAH. Screening for G6PD activity and mutations in the *G6PD* gene may provide early detection of individuals predisposed to PAH.

## Introduction

PAH characterized by a decrease of pulmonary artery diameter caused by abnormal proliferation of vascular wall cells. As a compensatory mechanism, the heart’s right ventricle is hypertrophied and blood pressure in the pulmonary arteries is increased. Oxygen deprivation manifests itself only at the later stage of the disease, shortage of breath, fatigue, and dizziness are all common clinical markers. As the disease progress further, heart failure is inevitable. Current treatments for PAH are unable to stop disease progression, and life expectancy is <5 years since diagnosis [1]. The only option that proved to be efficient is lung transplantation which is the last resort and has its complications. Understanding of the underlying mechanism of PAH would lead to the development of novel therapeutics.

PAH is a multi-hit disease, and in many cases genetic factors that contribute to PAH are unknown. One of the key genes affected in PAH is Bone Morphogenetic Protein Receptor Type 2 (BMPR2), a member of the TGFβ receptors superfamily. In the same time, the penetrance is only about 10–20% estimated by studies of large families with BMPR2 mutation carriers [2]. However, in familial PAH BMPR2 mutation accounts for up to 70% of cases [3].

Several other genes were found to be associated with PAH. They represent disparate pathways: prostaglandin signaling, nitric oxide biosynthesis and sensing, calcium homeostasis, and Wnt signaling [4, 5]. Inhibition of mitochondrial function due to mutations in SIRT3 is playing a role in PAH as it was shown in the study by Paulin et al. [6]. Carriers of sickle cell disease and thalassemia were shown to have an increased incidence of PH [7–10]. Anemia and increased hemolysis characterize both of these diseases. Since hemolysis is associated with PH, hemolysis-associated pathways need to be considered in the development of the pathology of PAH[11].

G6PD is a major producer of NADPH, which is in turn used for reduction of oxidized glutathione (GSSG). G6PD is the only producer of NADPH in erythrocytes besides 6-phosphogluconate dehydrogenase which is downstream of G6PD and therefore G6PD-dependent. G6PD deficient individuals are prone to acute hemolytic anemias triggered by a number of drugs or food. It was shown in a mouse model that intravascular hemolysis leads to a decrease in nitric oxide (NO) bioavailability due to NO scavenging by free hemoglobin which in turn causes vasoconstriction [12]. Another consequence of intravascular hemolysis is platelet activation and thrombosis. All of the mentioned above events are promoting PAH [13]. A clinical report connects G6PD deficiency with PAH [14]. The present study aims to screen PAH patients for the abnormalities in G6PD activity.

## Materials and methods

### Human subjects

The cohort consisted of patients with a diagnosis of Group I PAH including exercise-induced PH (PAH group, N = 22) as well as patients initially suspected of PAH, but later, diagnosed with other non-PAH conditions (non-PAH group, N = 8). Additional four patients in non-PAH group (N = 12 total) and 62 in PAH group (N = 84 total) were used for RNA-seq analysis, however, they were excluded from other studies because red blood cells were not collected for those patients. All patients underwent right heart catheterization (RHC). Basic clinical data was available through the electronic medical record and collected in a retrospective fashion for all patients, selecting closest clinical data to the date of the most recent RHC. All patients were prospectively recruited from the University of Arizona and provided written consent to participate in this study with the approval of the UA institutional human subjects review board (IRB N1100000621).

### G6PD activity measurements in Red Blood Cells (RBC)

Blood was collected using EDTA method and RBC fraction separated from plasma by centrifugation. RBC pellets were frozen and kept at -80C. Aliquots of RBC pellet were diluted in PBS and used for G6PD activity measurements using fluorometric activity assay kit (Abcam, ab176722). Protein concentration measured using BCA Protein Assay Kit (ThermoFisher Scientific). G6PD activity is usually presented in units per milliliter of blood or per gram of hemoglobin. Since at the time of experiment only frozen RBC pellets were available, G6PD enzymatic activity has been measured in arbitrary units, the rate of enzyme activity adjusted to total protein concentration and presented as a percentage in relation to the control group mean. It is referred to in the text as an adjusted activity. In order to detect changes in G6PD enzyme efficiency normalized activity was calculated; for that G6PD activity was divided by the G6PD concentration in the sample, which in turn, was measured by Western blot.

### RNA/DNA sequencing and gene expression analysis

Total RNA was extracted from PBMC cells using MagMax-96 Total RNA Isolation Kit. (Applied Biosystems, cat. No. AM1830). RNA was eluted in 40 μl of supplied elution buffer. An additional DNase treatment was performed using the ZR-96 RNA Clean & Concentrator kit (Zymo Research, cat. No. R1080) as per manufacturer’s instructions. All RNA samples were analyzed on the gel (Agilent 2200 Tapestation) and quantified using the Quant-iT RiboGreen RNA assay (Invitrogen, cat. No. R11491).

Only samples with RIN ≥6 were used for library construction. Sequencing libraries were prepared using SENSE mRNA-Seq library prep kit V2 (Lexogen cat. No. 001.96) as per instructions. Input amount of total RNA per sample was 50 ng. Libraries were PCR amplified for 20 cycles and quantified using Quant-iT PicoGreen dsDNA assay (Invitrogen, cat. No. P7581). All libraries were pooled together at equimolar concentrations, and the final pool was re-quantified using the KAPA Library Quantification Kit (KAPA Biosystems) before loading on the sequencer. The average insert size in a pool was 267 nt. Sequencing was carried out on NextSeq 500 (Illumina), 2x75 nt reads, using high output option. Approximately 400 million reads were obtained per output.

The normalized expression for the G6PD gene was calculated for each sample for the transcript ENST00000621232.4. RNAseq data was used for detection of mutation in the G6PD gene. Identified mutations were confirmed by Sanger sequencing of the corresponding genomic regions using DNA extracted from PBMC. DNA was extracted from 100ul of PMBC using QIAmp DNA blood mini kit (Qiagen). PCR amplification performed using high fidelity polymerase (Platinum SuperFi 2X master mix, Invitrogen) and fragments purified using MinElute PCR purification kit (Qiagen). All primer sequences could be found in the S1 Table.

### Western blotting

RBC pellets were diluted in PBS (1 in 100), and aliquots were mixed with reducing Laemmli buffer. The same dilutions were used for the protein concentration measurement using BCA method. SDS-PAGE gels (4–20%) and Western blots were performed using equipment and reagents from Bio-Rad according to manufacturer’s recommendations. Western blots performed using antibodies against G6PD (sc-373886, Santa Cruz Biotechnology), transferrin (A1448, ABclonal) and transferrin receptor (13–6800, Invitrogen).

### Statistics

Statistical analysis was performed using GraphPad Prism version 5.01 (GraphPad Software, San Diego, CA). Outliers were identified with Grubbs' test using GraphPad outlier calculator (Alpha = 0.5) at https://www.graphpad.com/quickcalcs/Grubbs1.cfm. At least two measurements were performed for each experiment; their average is calculated and used for plotting. For analysis of groups, mean and SEM are calculated and indicated on the plots. The significance is calculated using unpaired t-test and two-tailed P value calculated with 95% confidence interval. Correlation analysis performed using GraphPad. Pearson or Spearman correlations are calculated, and r values, as well as p values, are indicated.

## Results

Twenty-two patients with PAH were studied. Eight control subjects were non-PAH patients with other lung pathologies. Clinical and laboratory assay data are presented in Table 1. The following parameters were measured: Cardiac Output (CO), mean Pulmonary Arterial Pressure (mPAP), Pulmonary Vein Resistance (PVR), B-type natriuretic peptide (BNP) which is a heart failure protein marker, G6PD activity and transferrin receptor concentration in RBC pellets adjusted to total protein concentration and level of mRNA expression for *G6PD* gene in PMBC.

**Table 1:** Demographic and clinical characteristics of patients with and without PAH. Clinical and lab data for PAH patients and control non-PAH subjects. IPAH–idiopathic PAH, APAH–associated PAH, exPH–exercise-induced PH, drPAH–drug-induced PAH. CO—Cardiac Output (CO), mPAP—mean Pulmonary Arterial Pressure, PVR—Pulmonary Vein Resistance, BNP—B-type natriuretic peptide. TfR–transferrin receptor (CD71). G6PD activity and transferrin receptor concentration in RBC pellets are adjusted to total protein concentration and presented in % to the control group mean.

ID	age	sex	PAH type	mPAP	PVR	CO	BNP	mRNA	G6PD activity	TfR	Transferrin
1c	67	♂	non-PAH					1127	99	0.06	11
2c	67	♀	non-PAH					1316	99	1.74	180
3c	73	♀	non-PAH	17	2.2	3.9	41	1099	87	1.20	441
4c	34	♀	non-PAH	15	1.1	7.6	10	1798	86	0.02	99
5c	61	♀	non-PAH	18	1.9	5.3	16	1680	84	1.05	441
6c	77	♀	non-PAH	31	2.3	6.6	88	2158	120	1.92	94
7c	19	♂	non-PAH	13	0.7	6.7	28	1094	114	1.69	113
8c	85	♂	non-PAH	26	2.7	6.0	212	932	112	0.31	103
9	31	♀	APAH	95	17.5	4.7	60	763	154	18.44	187
10	79	♀	APAH	26	4.3	4.2	287	812	111	0.01	108
11	71	♀	exPAH	20	2.0	5.4	970	2394	95	0.28	38
12	63	♂	APAH	33	3.8	7.3	37	637	28	0.35	100
13	66	♂	exPAH	20	1.4	3.6	61	1816	41	2.89	475
14	45	♂	drPAH	53	7.2	5.4	11	1088	118	0.64	134
15	62	♀	APAH	41	3.9	7.0	226	1186	79	0.09	65
16	65	♀	exPAH	14	2.1	3.3		1521	128	0.01	213
17	76	♂	IPAH	43	3.7	6.9	123	943	146	0.97	140
18	63	♂	APAH	35	4.3	4.5	76	1722	116	0.10	237
19	70	♀	exPAH	20	2.4	6.3	19	1680	126	0.52	480
20	60	♀	exPAH	19	1.5	5.3	64	2245	122	0.47	228
21	51	♂	drPAH	54	10.6	4.6	1708	1864	124	0.22	127
22	49	♀	APAH	28	4.1	4.9	55	1863	73	1.08	48
23	38	♀	IPAH	41	3.4	7.8	182	2510	199	3.90	312
24	53	♂	APAH	30	3.3	5.8	385	2322	157	2.70	59
25	65	♀	APAH	34	3.2	6.0	101	2954	156	3.70	209
26	54	♀	IPAH	26	3.4	5.8	50	3411	133	4.18	113
27	52	♀	APAH	48		11.6		3249	190	48.35	103
28	44	♀	APAH	31	1.4	7.7	125	1841	32	19.87	70
29	67	♂	IPAH	58	5.1	7.8	993	1974	193	0.79	304
30	42	♀	IPAH	41	5.7	5.1	28	1842	167	3.47	291

### G6PD activity in RBC from patients

**Fig 1:**
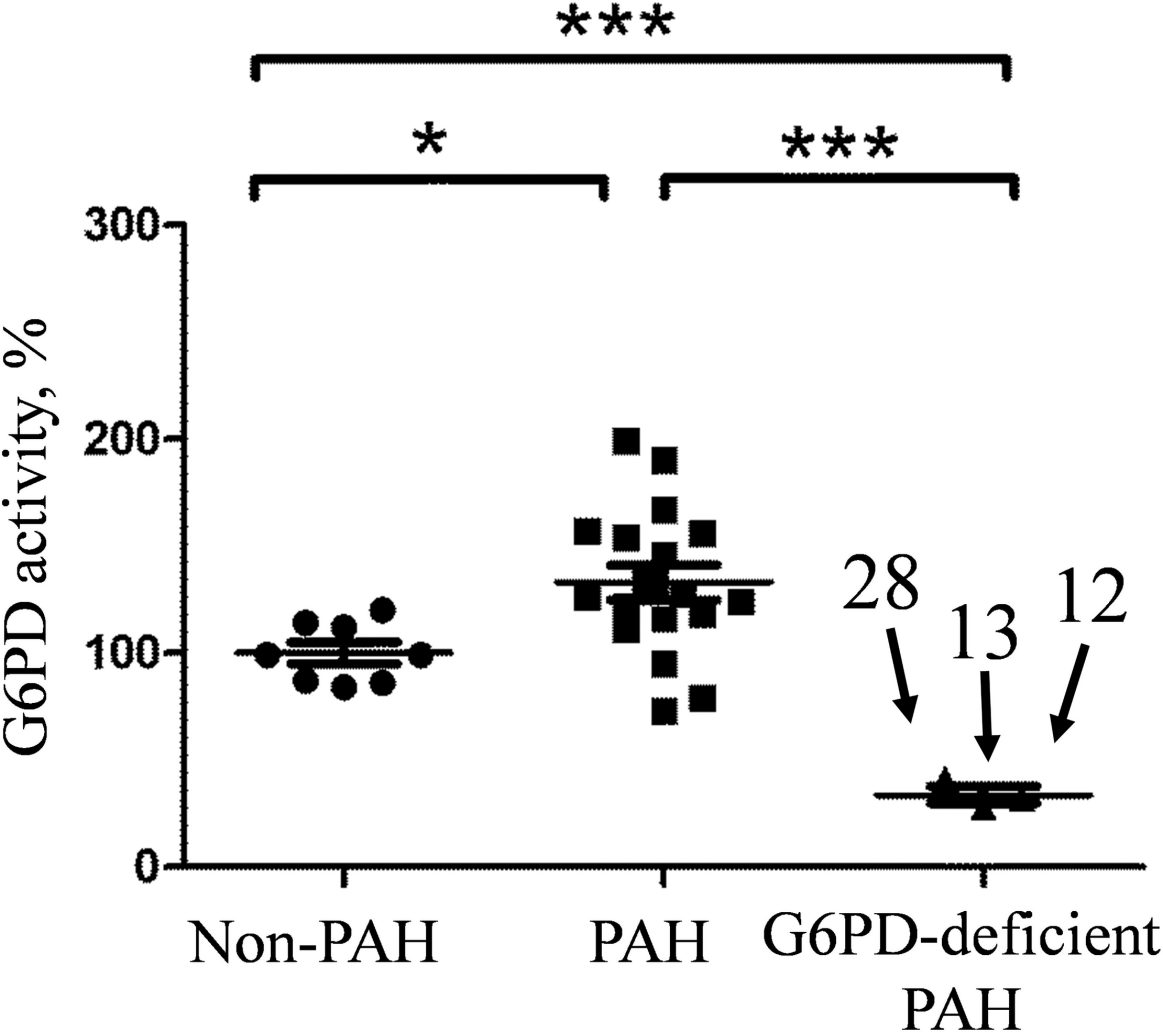
G6PD activity in RBC pellets. Activity is normalized to protein concentration and presented in relation to the non-PAH group mean. Measurements are done in duplicates and the average of two for each sample is plotted. Three samples with the lowest G6PD activity form a separate group. Unpaired t-test is used for pairwise comparison of groups. *p = 0.015, *** p = 0.0001.

G6PD enzymatic activity was measured in the RBC pellets from patients. Three out of twenty-two PAH patients were deficient for G6PD activity in their RBC pellets. Their activities were 28% (N12), 32% (N28) and 41% (N13) of non-PAH. These three samples form a separate group with a mean that is significantly different from non-PAH. Two more patients demonstrated decreased G6PD activity: 73% for N22 and 79% for N15. The rest of the PAH samples had a broad range of G6PD activity with the group mean value being significantly higher than the non-PAH group. (Fig 1).

**Fig 2:**
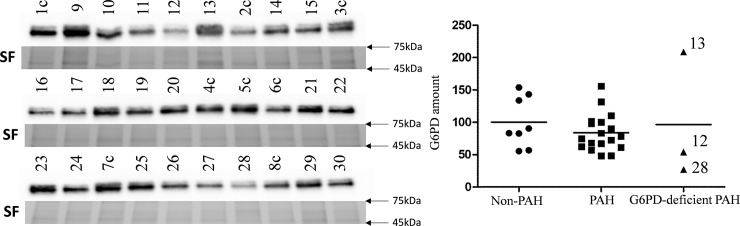
Western blot analysis of RBC pellets for G6PD protein. 40 μg of protein was loaded for each sample. Stain-free (SF) gel used for normalization (full gels available on S1 Fig). Values are in % to the non-PAH group mean. Three G6PD-deficient samples plotted separately.

Amount of G6PD protein in RBC pellets was measured by Western blot analysis (Fig 2).

Out of three samples with low G6PD activity two also showed a low level of G6PD protein (samples N12 and N28) while one had a high amount of G6PD protein.

Using data from Western blot, G6PD activity was normalized to the amount of G6PD protein. After normalization, the differences between groups became statistically insignificant as expected since the normalized activity is the activity per molecule of enzyme. However, some differences in normalized activity were present. G6PD deficient samples N12 and N28 showed near normal G6PD activity after normalization. Only one sample (N13) demonstrated a significant decrease in activity after normalization; it was 32% of the non-PAH group mean.

### G6PD gene expression in PBMC

**Fig 3:**
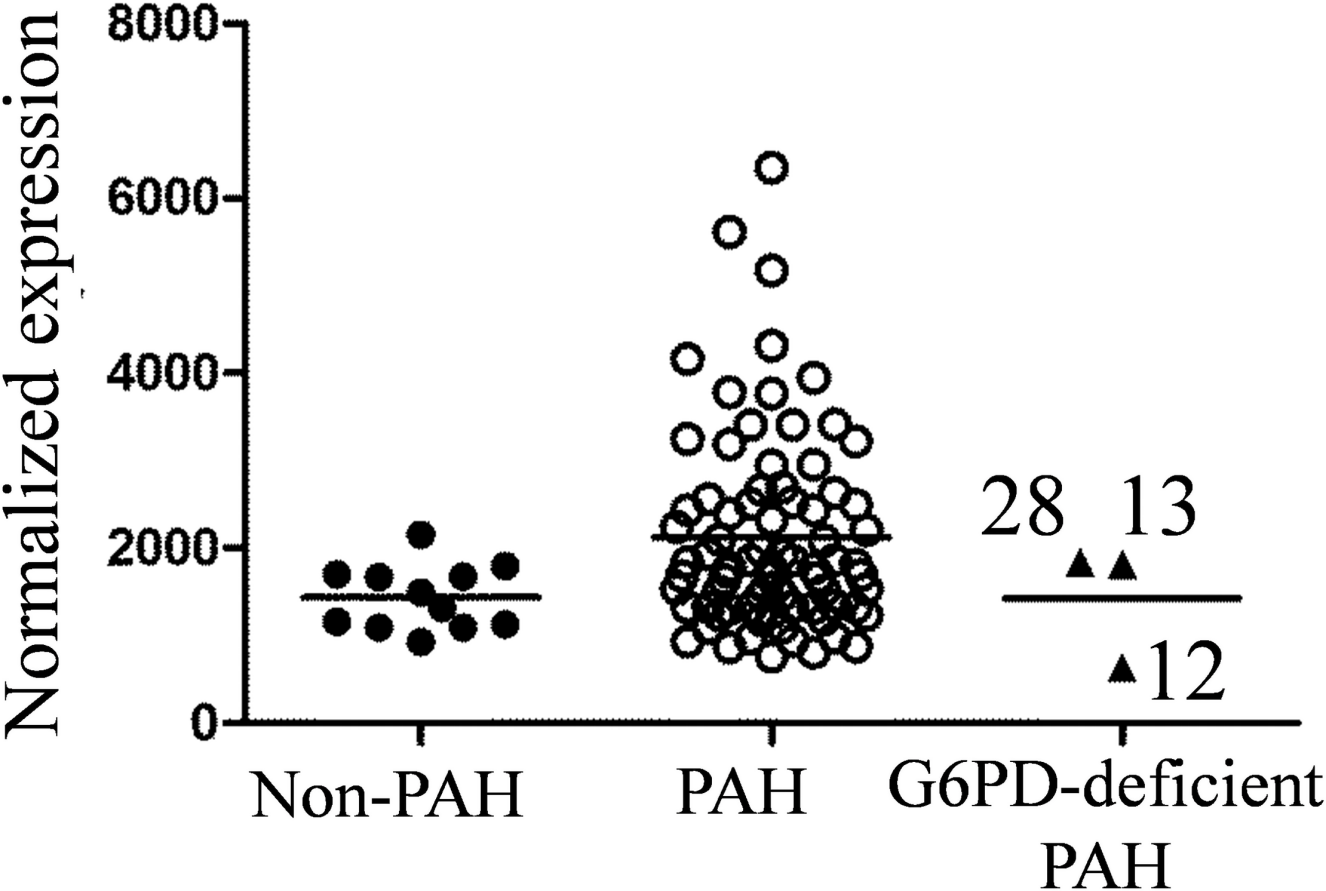
G6PD mRNA expression in PBMC measured by RNAseq. Three G6PD-deficient samples plotted separately.

*G6PD* gene expression level was measured in PBMC by RNAseq, and normalized expression is presented in Fig 3.

**Fig 4:**
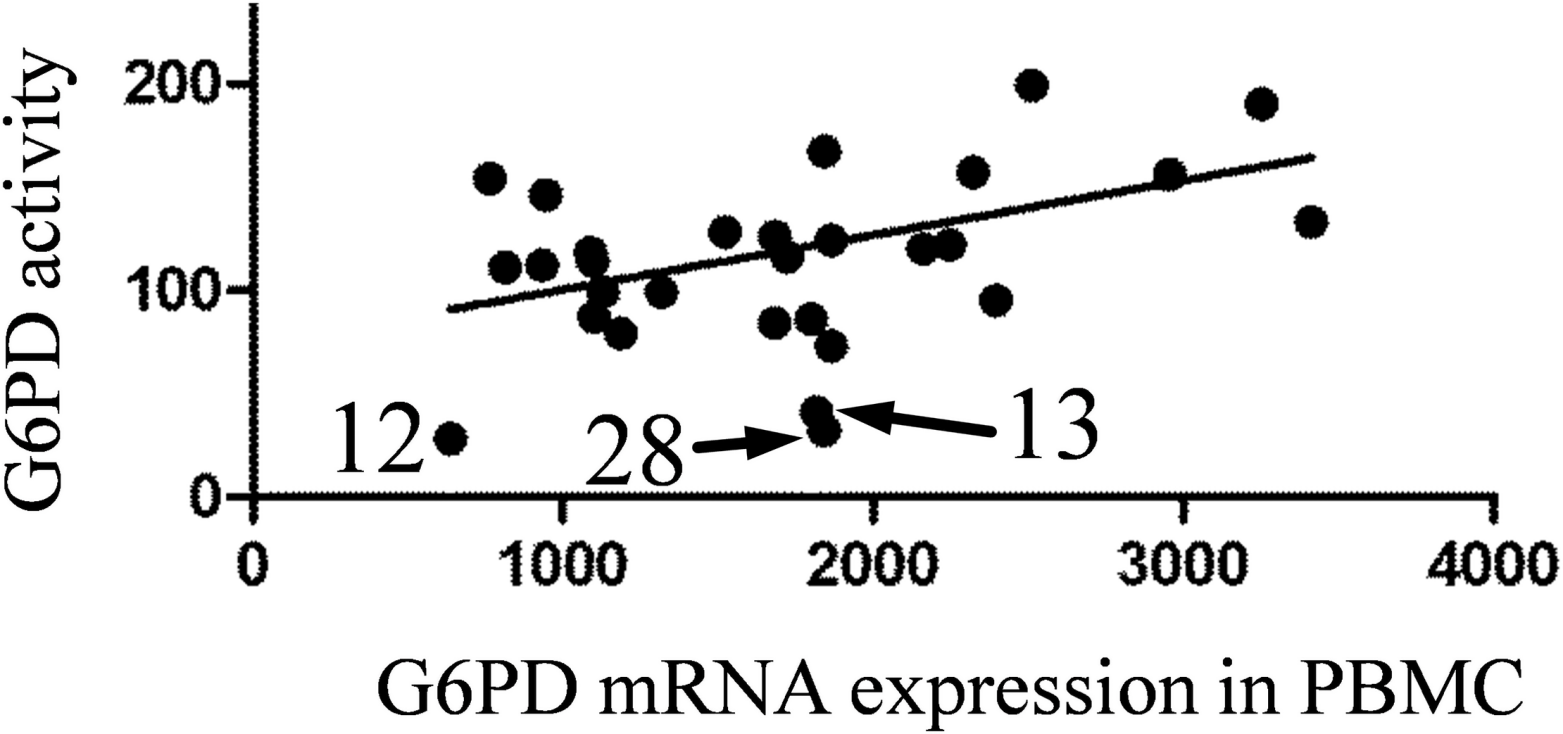
Correlation of G6PD activity (adjusted to total protein concentration) in RBC pellets and G6PD mRNA expression in PBMC. Spearman r = 0.4, P = 0.028. Three G6PD-deficient samples are indicated.

Variation in *G6PD* mRNA expression is high across PAH samples, and their expression means is higher than in the non-PAH group. Sample N12 (from G6PD deficient patient) showed the very low level of *G6PD* mRNA expression in PBMC (44% of the non-PAH group mean). The level of *G6PD* gene expression in PBMC is positively correlated with the G6PD activity in RBC (Fig 4).

### G6PD mutation analysis

Protein coding region of the gene was analyzed. Mutations revealed in RNAseq experiment were confirmed by Sanger sequencing of PCR-amplified genomic DNA. Only missense mutations are reported (Table 2). Sequencing of G6PD mRNA revealed three PAH samples with missense mutations. The mutation in patient N28 was described previously and is known to be associated with a G6PD deficiency, our results indicate 68% drop in G6PD activity in this individual. Another mutation was also reported to be associated with mild G6PD deficiency, and we also found a 21% drop in activity in patient N15. Patient N16 carried a mutation that is not associated with a G6PD deficiency and showed an increase in G6PD activity in RBC pellet.

**Table 2:** Missense mutations of G6PD found in PAH patients. G6PD activity reported in % to control group mean.

ID	Position on cDNA	Amino acid change	Exon number	zygosity	Sex	SNP ID	G6PD activity % (reported previously)	G6PD activity % (this study)	PAH type
15	c.376 AAT>GAT	Asn 126 Asp	5	het	♀	rs1050829	80–100 (12–14)	79	Associated PAH
16	c.582 GAC>GAG	Asp 194 Glu	6	het	♀	rs145247580	90–100 (15)	128	Exercise induced
28	c.871 GTG>ATG	Val 291 Met	9	het	♀	rs137852327	50–70 for het♀ (17)	32	Associated PAH

### Estimation of reticulocyte count in RBC pellets

**Fig 5:**
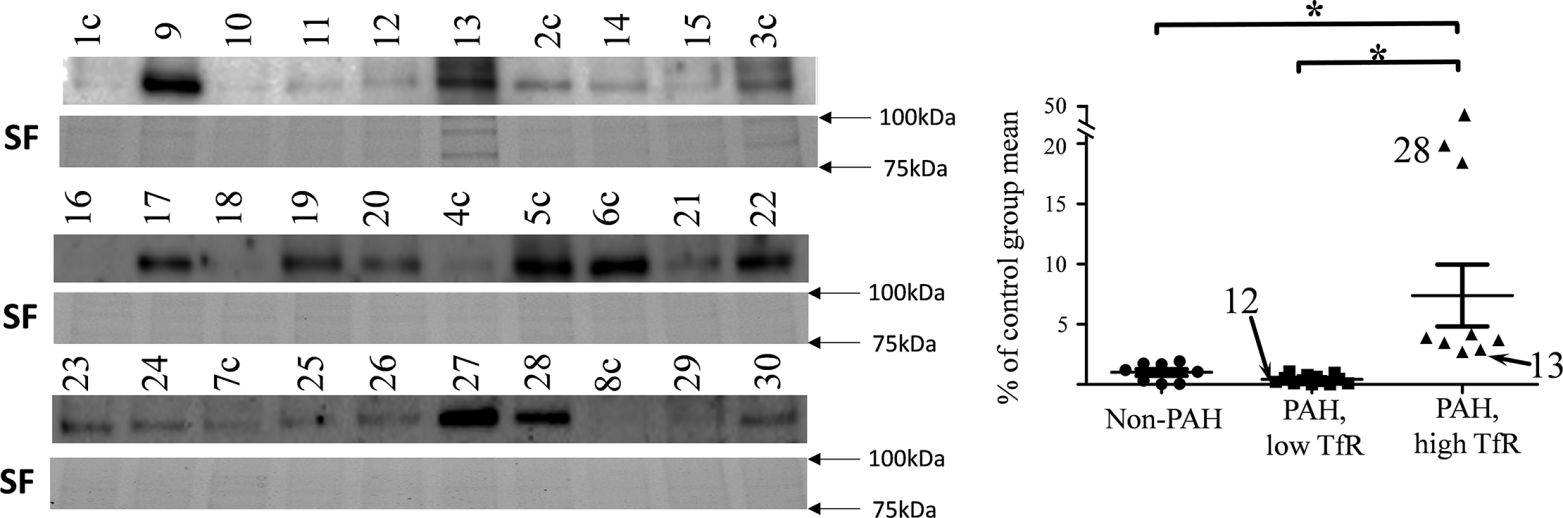
Western blot for transferrin receptor (TfR), CD71 protein in RBC pellets. The signal is normalized to the stain free (SF) gels and data presented as % of the non-PAH group mean (full gels available on S2 Fig). PAH samples formed two distinctive groups; one with high levels of CD71 and one with low levels of CD71. Three G6PD-deficient samples are indicated.

Since only frozen RBC pellets were available for analysis, Western blot analysis for reticulocyte marker, transferrin receptor protein (TfR, CD71) was used as a marker for reticulocyte count (Table 1). An increased amount of TfR was found in nine out of twenty-two PAH samples (Fig 5).

**Fig 6:**
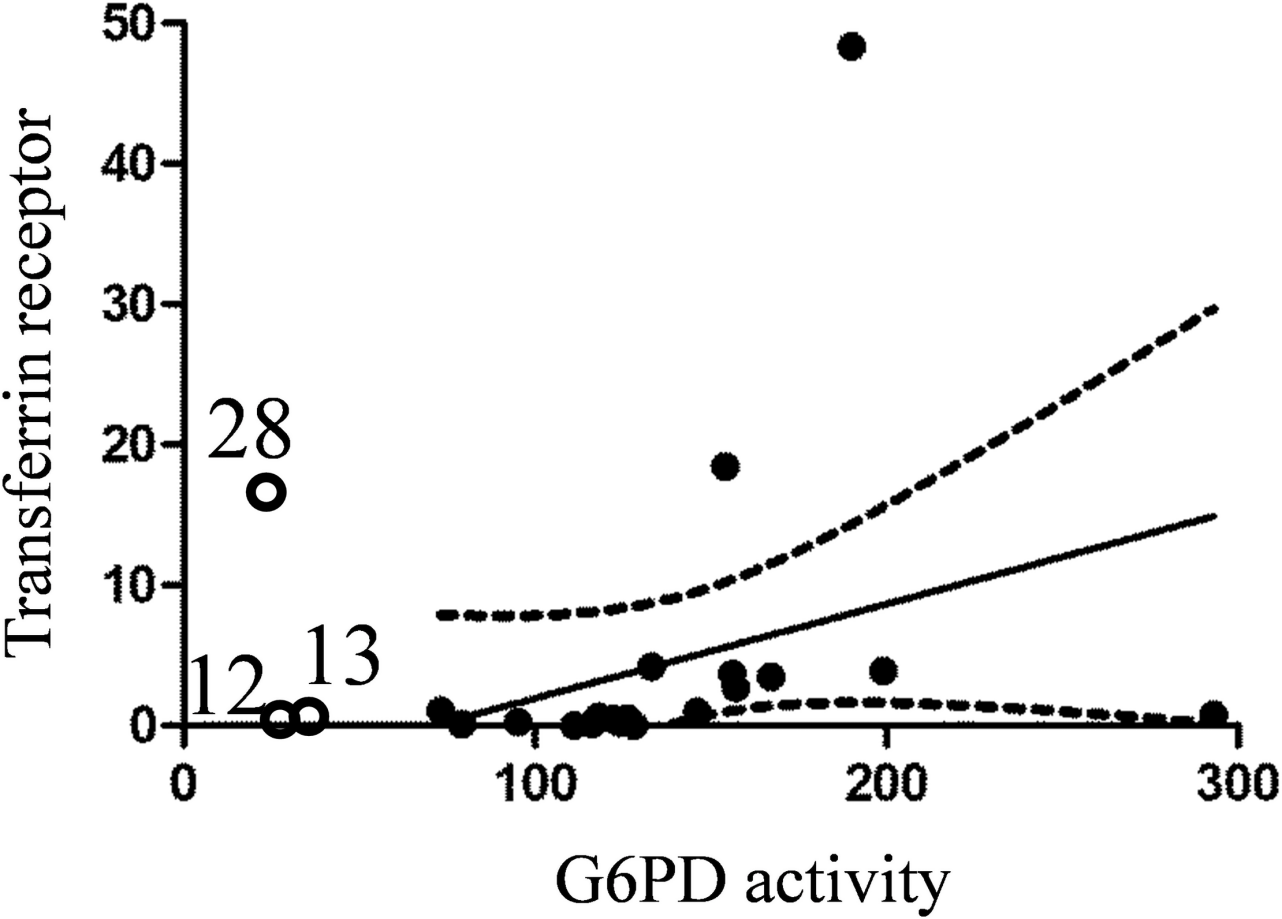
Correlation between G6PD activity and transferrin receptor (CD71) concentration in RBC pellets for PAH patients only. Spearman r = 0.65, p = 0.02. 90% confidence band is indicated by dotted line. G6PD-deficient samples (N12, N13 and N28) were not used for analysis but indicated on the graph.

It is in agreement with a previously reported increase of erythropoiesis in PAH patients; Potoka et al. found that erythropoietin level was 7.9–13.6 times higher than in healthy individuals [15]. Reticulocyte count increase is a well-known compensatory mechanism for oxygen deprivation, and this could explain high G6PD activity detected in RBC pellets of PAH patients. A positive correlation between TfR concentration and G6PD activity was found (Fig 6).

Since reticulocytes have the significantly higher amount of G6PD protein than erythrocytes, such an increase in reticulocyte count affects total G6PD activity.

G6PD-deficient patient N28 showed 20 times increase for reticulocyte marker (CD71) which didn’t restore total G6PD activity in blood to the normal level. Another G6PD-deficient patient (N13) has 3 times more CD71 than controls. Level of CD71 in a third G6PD-deficient patient (N12) is within the non-PAH group range.

### Measurement of transferrin protein

**Fig 7:**
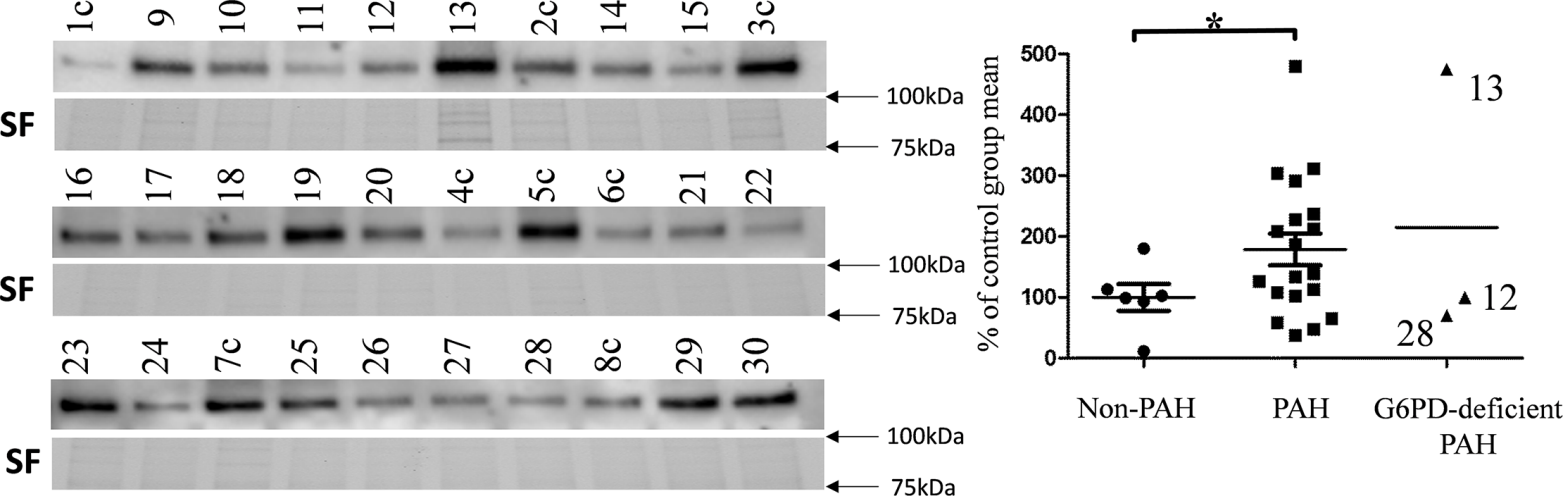
Western blot for transferrin protein in RBC pellets. Signal is normalized to stain free (SF) gels and data presented as % of the non-PAH group mean (full gels available on S3 Fig). Two outliers were removed from the non-PAH group after performing an outlier test. Significance in values between non-PAH and PAH groups is confirmed in unpaired t test with Welch’s correction. p = 0.03. Three G6PD-deficient samples plotted separately.

Amount of transferrin protein is an indicator of body response to anemia. We measured the amount of transferrin protein in RBC pellets in Western blot (Fig 7).

The amount of transferrin for SNP-carrying patient N16 is increased ~2 times compared to non-PAH while G6PD activity in blood is only 28% higher than in the non-PAH group. Similarly, N19 showed nearly 5 times increase in transferrin while G6PD activity was only 26% higher.

**Fig 8:**
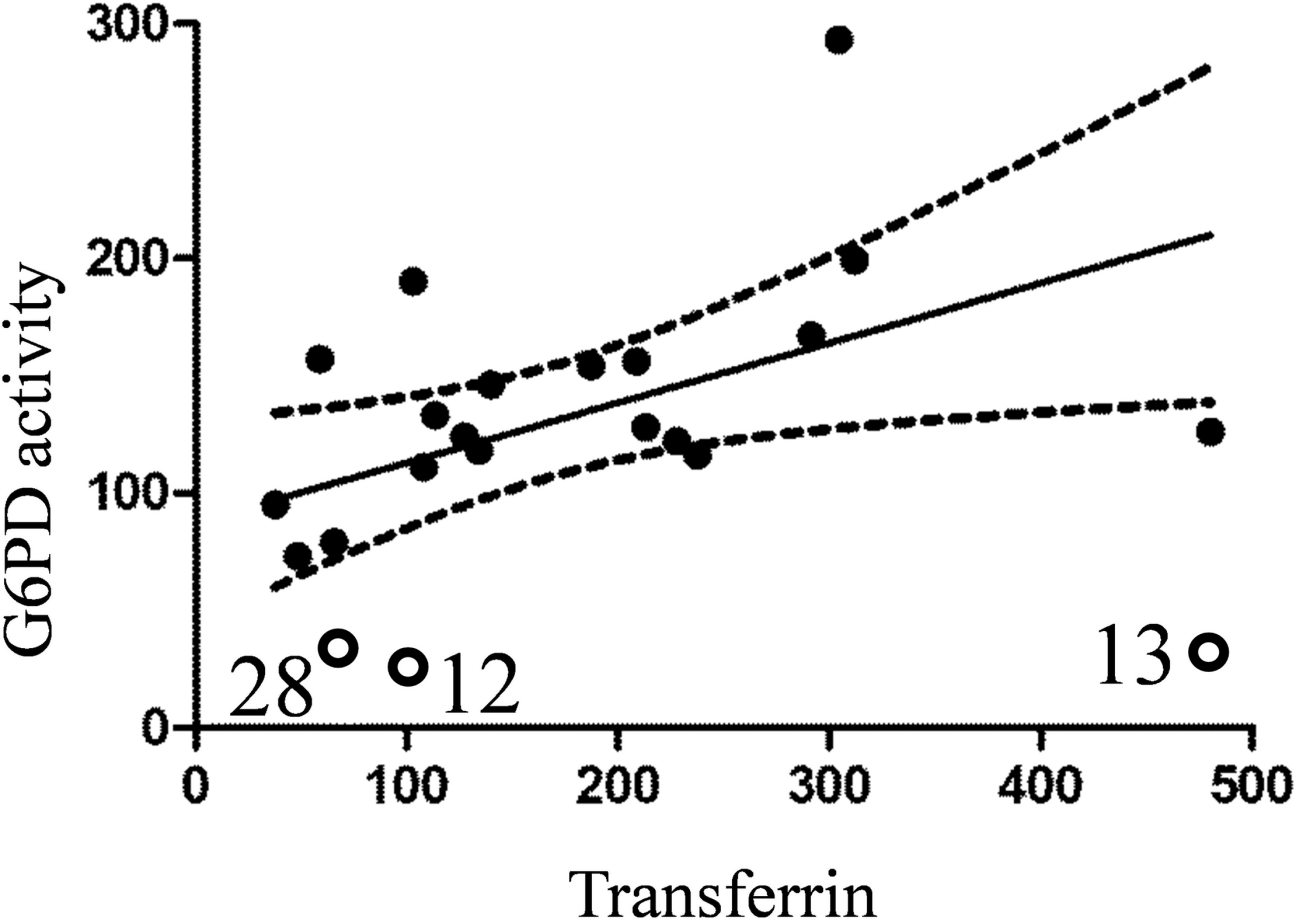
Correlation between G6PD activity and transferrin concentration in RBC pellets for PAH patients only. Spearman r = 0.474, p = 0.04. 90% confidence band is indicated by dotted line. G6PD-deficient samples (N12, N13, and N28) were not used for analysis but indicated on the graph.

Two other SNP-carrying patients (N15 and N28) showed a slight decrease (~30%) in transferrin. Finally, high level of transferrin in patient N13 (475%) did not result in restoration of G6PD activity which was 41% of the non-PAH group mean. A positive correlation between transferrin concentration and G6PD activity was found (Fig 8).

### Correlation between clinical parameters and G6PD activity

**Fig 9:**
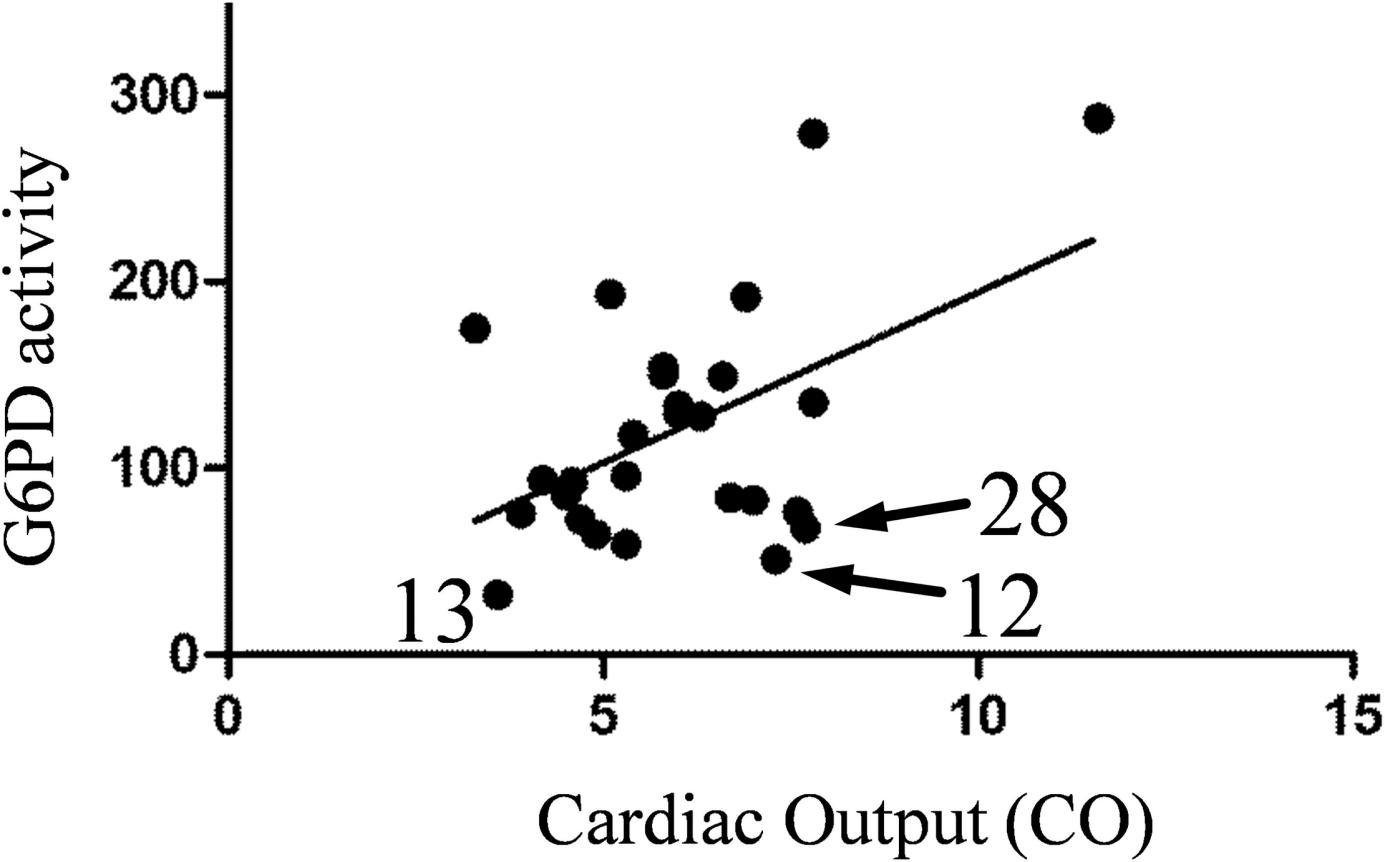
Correlation of normalized G6PD activity in RBC pellets and Cardiac Output. Normalized activity is calculated by dividing G6PD activity by G6PD concentration measured in Western blot. Pearson r = 0.5, P value (two-tailed) = 0.007.

Analysis of clinical data revealed some weak but significant correlations with G6PD activity. Cardiac Output (CO) is the measure of heart function during the PAH progression, and lower CO corresponds to more severe PAH. Normalized G6PD activity is positively correlated with Cardiac Output (Fig 9).

It means that lower enzymatic activity of G6PD correlates with a disease progression. Other hemodynamic and clinical parameters did not significantly correlate with G6PD activity significantly in the studied small cohort due to the size of the cohort studied.

## Discussion

As patients with hemolytic anemias show a higher prevalence to develop pulmonary hypertension than the general population, it suggests the involvement of hemolysis in the pathobiology of the disease. This yields the question, is pulmonary hypertension also associated with other types of predispositions to hemolysis? For example, mutations in the *G6PD* gene that leads to decreased activity of the protein in erythrocytes can induce underlying hemolysis in people. The *G6PD* gene is located on chromosome X, and about 160 missense mutations are known for this gene. The majority of these mutations are affecting G6PD’s enzymatic activity or stability. In the present study, a small cohort of PAH patients (N = 22) was screened for the presence of mutations in the *G6PD* gene. Sequencing of G6PD mRNA revealed three PAH samples with missense mutations (Val291Met, Asn126Asp, Asp194Glu).

Asn126Asp mutation (rs1050829) has been reported previously as the cause of 10–20% decrease in G6PD activity [16, 17] while others found no difference in G6PD activity for this protein variant [18]. We have found 21% decrease in G6PD activity from a heterozygous female PAH patient that carries this mutation (N15). We explain this decrease in G6PD capacity by enzyme stability/lifetime because it’s normalized activity (activity recalculated to the amount of G6PD in the sample), was not different from controls. The severity of disease for this patient manifested itself in high mPAP around 40mmHg and high level of BNP.

Asp194Glu mutation (rs145247580) was found in heterozygous female PAH patient (N16). Asp194Glu mutation was originally reported without any data for G6PD activity. Recently G6PD activity was reported for one carrier of this mutation (heterozygous female) which was 9.2 U/g Hb. The normal range for G6PD activity is between 9.9 to 16.6 which makes this variant slightly deficient [19]. We have not detected any changes in G6PD activity for this sample under our experimental conditions. Interestingly, this patient was diagnosed with exercise-induced PH which showed increased pulmonary pressure during exercise, at rest, this patient had normal mPAP. Increased ROS production during exercise can trigger the underlying G6PD mutation and produce hemolysis [20]. In the future, the possibility of exercise-induced hemolysis due to G6PD deficiency and possible connections with PAH need to be explored.

Val291Met mutation (rs137852327) known as Viangchan have been reported to severely affect G6PD activity in hemizygous males (4–12 times) and moderately affect G6PD activity in heterozygous females (0.7–2 times)[21]. Mutation is affecting the stability of the enzyme [22]. We have found a 3-fold decrease in G6PD activity in the heterozygous female that carries Viangchan mutation (N28). The amount of G6PD protein in RBC is indeed dramatically decreased compared to controls (Fig 2). RNAseq showed the prevalence of mutated over WT transcript in PBMC (82:18 ratio). A well-known phenomenon of asymmetrical X chromosome inactivation is a plausible explanation for our findings. Since PBMC and RBC shared the same origin, we could speculate that immature erythrocytes have had the same ratio. In spite of a significant decrease in G6PD activity, this patient has a mild form of PAH with mPAP around 30mmHg.

We have also analyzed G6PD activity in RBC pellets and *G6PD* mRNA expression in PBMC in PAH patients that were not associated with a missense mutation in the G6PD gene. Oxygen deficiency characterizes PAH during the uncompensated phase. Thus, there is no surprise that the amount of reticulocytes in many PAH patients is elevated. This fact can explain the elevated level of G6PD activity (Fig 1) in the majority of PAH patients. Since detection of G6PD deficiency in PAH patients could be masked by increased hematopoiesis, other measurements such as G6PD protein concentration or reticulocyte markers are advisable.

G6PD activity normalized to the amount of G6PD protein is an important measurement of a functional and clinical significance as it is able to pinpoint abnormalities in NADPH production due to decreased enzyme efficiency or stability. It is expected that normalized activity would be similar for all samples as the difference in G6PD concentration does not vary between samples. Interestingly, it was not the case, and we saw two samples where normalized G6PD activity was ~3 times higher than in controls (N27 and N29). At the same time, two out of three G6PD-deficient samples (N12 and N28) showed near normal G6PD activity after normalization. Only one sample (N13) had significantly decreased activity after normalization. Even though we have not found mutations in the coding region of the gene, it is obvious that its enzymatic output is affected, but its cause is unknown. It worth noting that the organism tries to compensate for such inefficiency as we can see an increase in G6PD protein production (Fig 2).

Since erythrocytes are almost free of mRNA and *de novo* protein synthesis, we have measured mRNA expression in PBMC. *G6PD* mRNA level was elevated in PBMC for most of PAH samples. Again this fact could be explained by compensatory mechanisms and increased amount of reticulocytes that are co-isolated with PBMC. We have found a good correlation between *G6PD* mRNA level in PBMC and G6PD activity in RBC (Fig 4).

Low levels of G6PD mRNA in PBMC was detected for patient N12 (44% of the control group mean), and it correlated well with low-level G6PD protein in RBC. This patient showed ~3 fold decrease in G6PD activity (28% of the control group mean value). According to a recent study by Taniguchi et al. G6PD activity is correlated with the mRNA level [23]. We believe that it is a plausible explanation for an observed decrease in G6PD activity.

Another male (N13) with exercise-induced PAH showed decreased G6PD activity in spite of the elevated level of G6PD protein (Fig 2). Inefficiency in NADPH production was obvious since its normalized G6PD activity was significantly lower than in controls. Since no mutations were found in the coding region, the cause of G6PD deficiency is unclear. There are reports of posttranslational modifications that modulate G6PD enzymatic activity, such as acetylation on lysine 403 [24] and in the future cases like this need to be investigated for such modifications.

Interestingly, from all G6PD deficient PAH patients found in this study only one (N28) is a carrier of a serious mutation, Viangchan, and this patient had relatively mild PAH. At the same time, two other carriers of G6PD mutations developed more severe PAH while their mutations are considered to be mild.

Analysis of clinical data revealed some weak but significant correlations with G6PD activity. Cardiac Output (CO) is the measure of heart failure during the PAH progression, and lower CO corresponds to more severe PAH. Normalized G6PD activity is positively correlated with Cardiac Output (Fig 9). It means that higher enzymatic activity rate is able to decrease the disease progression and could be attributed to the increased G6PD activity in PAH patients. It is important to note that G6PD could play opposite roles in different tissues; low G6PD in erythrocytes leads to higher hemolysis but slightly lower than normal G6PD activity was shown to be associated with lower risk of cardiovascular disease.

## Conclusions

Five out of twenty-two PAH patients were found to be G6PD deficient. Three of them demonstrated a significant decrease in G6PD activity in RBC. In one case (patient N12) the amount of G6PD protein was low most likely due to a decreased level of mRNA expression in RBC progenitor cells. In the second case (N28) the amount of G6PD is decreased due to enzyme instability which in turn is caused by Val291Met missense mutation. In the third case (N13) G6PD deficiency was detected in spite of the high level of G6PD protein in RBC and G6PD deficiency was even more pronounced after normalization to the amount of G6PD protein. Since no mutation was detected in the coding region of the gene the cause of G6PD deficiency is not clear. Two more patients demonstrated a moderate decrease in G6PD enzymatic activity, one of them (N15) is a carrier of missense mutation. And finally, the carrier of another missense mutation (N16) has developed an exercise-induced PAH while no significant decrease in G6PD activity was found.

## Supporting information

S1 Fig Uncropped images for G6PD western blot membranes and Stain-free SDS-PAGE gels. (TIF)

S2 Fig Uncropped images for Transferrin receptor western blot membranes and Stain-free SDS-PAGE gels. (TIF)

S3 Fig Uncropped images for Transferrin western blot membranes and Stain-free SDS-PAGE gels. (TIF)

S1 Table List of primers used for *G6PD* gene amplification and sequencing. (XLS)
