# Supplementary figures and images for: New cases of Glucose-6-Phosphate Dehydrogenase deficiency in Pulmonary Arterial Hypertension
Source: PLoS One. 2018 Aug 30;13(8):e0203493. doi: 10.1371/journal.pone.0203493 (PMC6117081; doi:10.1371/journal.pone.0203493)

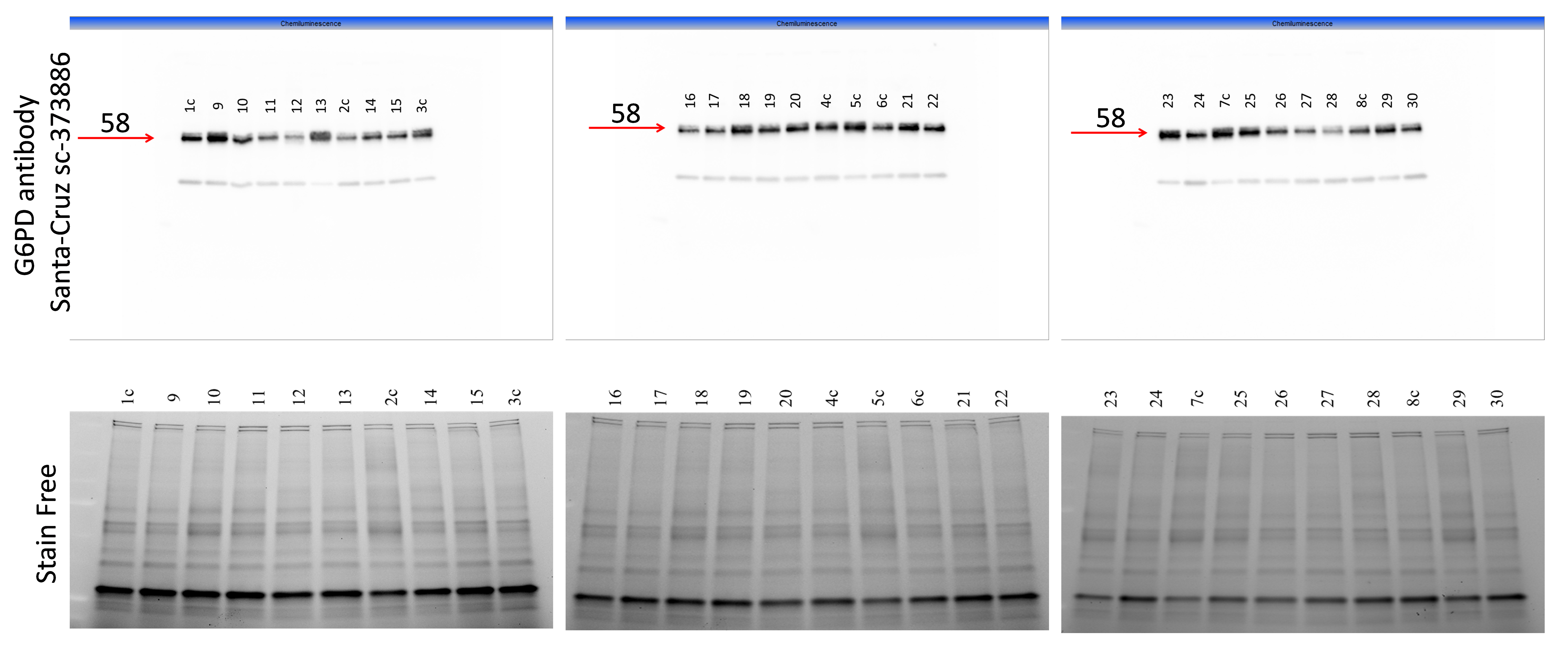

Supplement: S1 Fig — (TIF) [file pone.0203493.s001.tif]

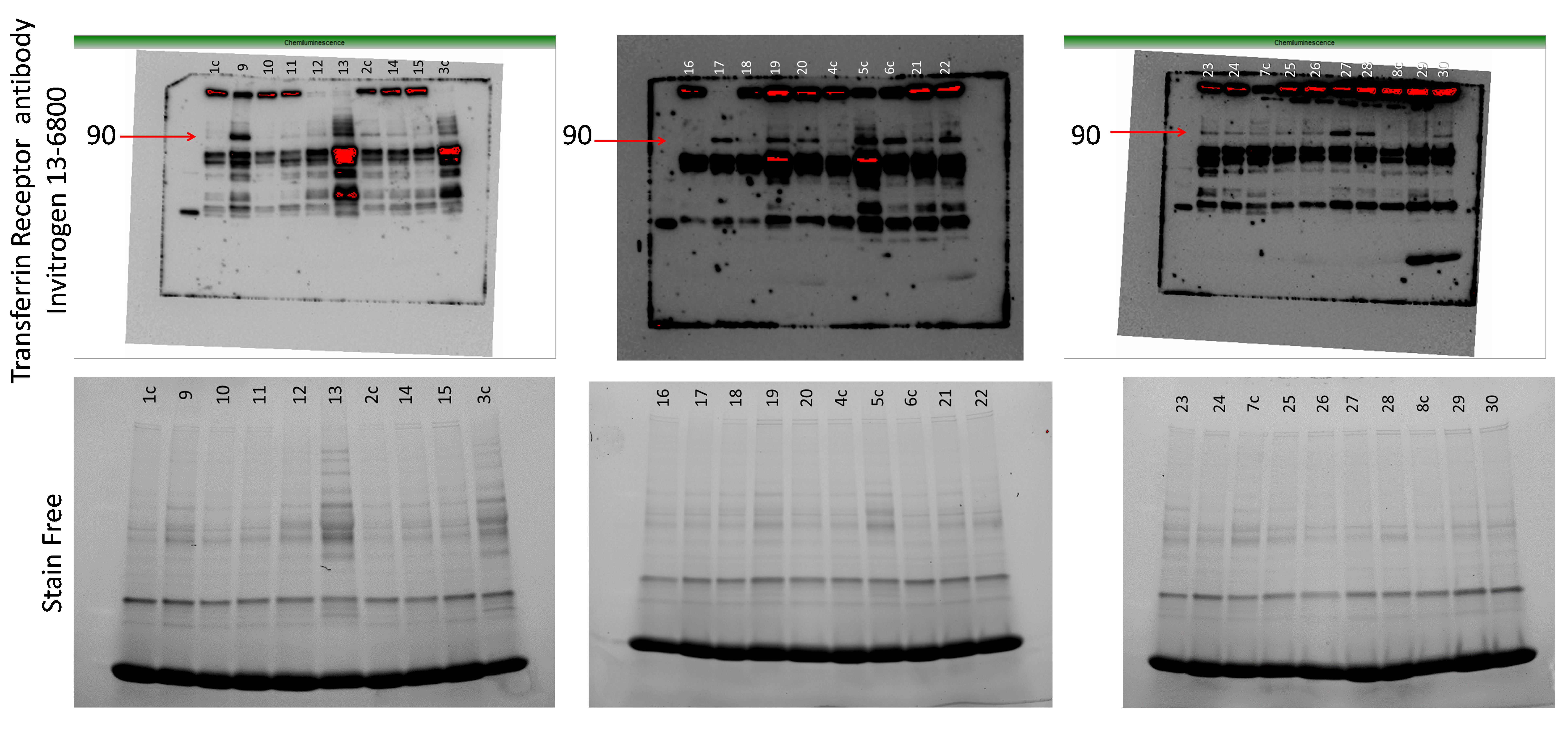

Supplement: S2 Fig — (TIF) [file pone.0203493.s002.tif]

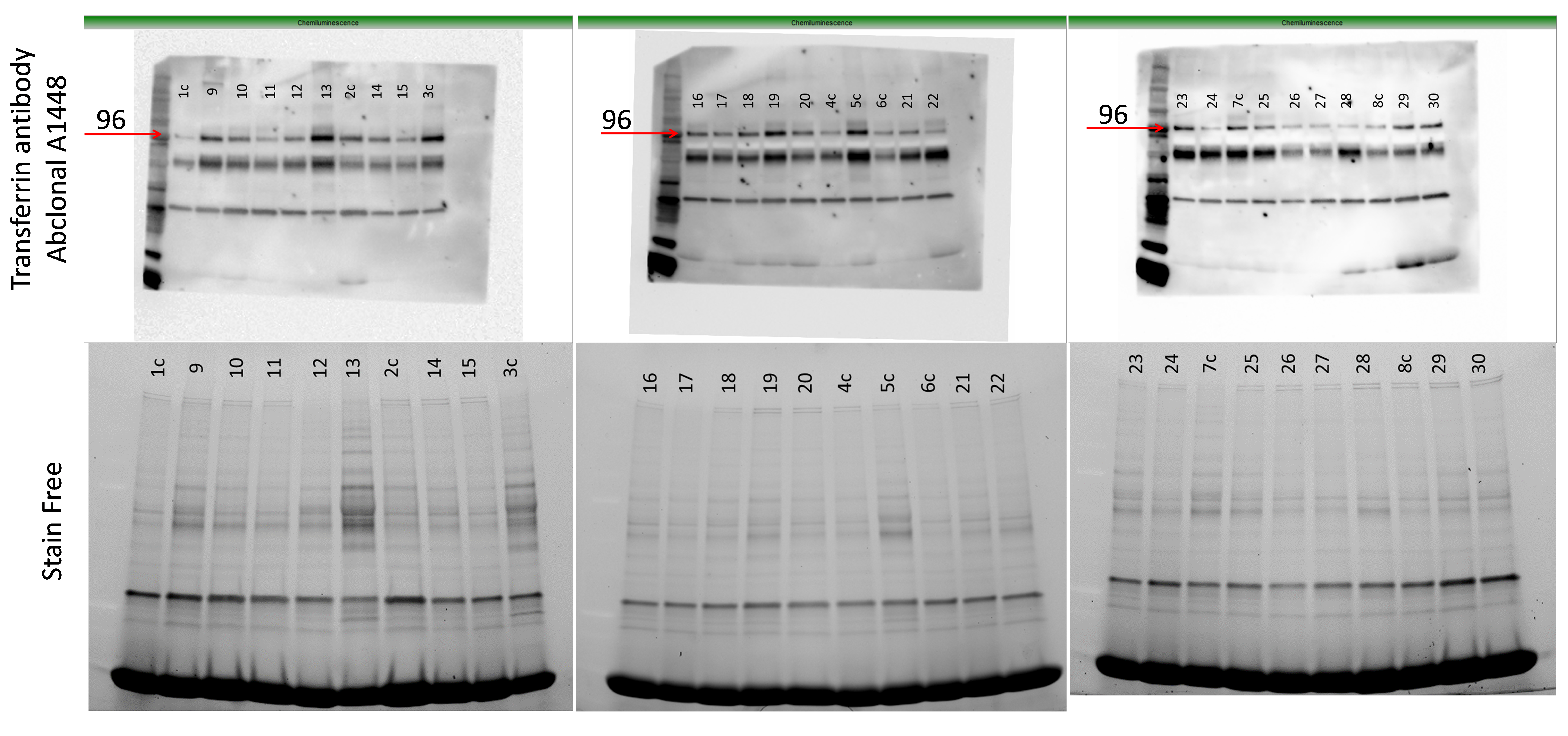

Supplement: S3 Fig — (TIF) [file pone.0203493.s003.tif]
